# Microbial succession and tissue-specific restructuring of bacterial and fungal communities during post-harvest onion bulb rotting

**DOI:** 10.3389/fmicb.2026.1776996

**Published:** 2026-02-20

**Authors:** Satish Kumar, Ram Dutta, Radhakrishna Auji, Goraksha C. Wackchaure, K. Jayalakshmi, Vadivelu Karuppaiah, Vijay Mahajan

**Affiliations:** 1 ICAR-Directorate of Onion and Garlic Research, Pune, India; 2 ICAR-National Institute of Abiotic Stress Management, Baramati/Pune, India

**Keywords:** amplicon sequencing, onion bulb rotting, onion microbiome, postharvest spoilage, tissue-specific microbiome

## Abstract

Despite being a relatively hardy bulb crop with a longer shelf life than many fresh vegetables, onions are susceptible to substantial postharvest losses due to microbial spoilage. This study used high-throughput amplicon sequencing to characterize the bacterial and fungal microbiomes associated with healthy (HB), mildly rotten (MRB), and severely rotten (SRB) onion bulbs. Microbial communities were analysed across three distinct bulb tissues comprising neck tissue (NT), outer scale (OS), and central tissue (CT), to generate stage-specific and tissue-specific microbiome profiles. The microbial community analysis based on over 2 million Illumina NGS reads revealed the presence of 85 bacterial OTUs and 53 fungal OTUs across nine bulb samples. Bulb deterioration was marked by pronounced microbial succession, with bacterial diversity increasing from healthy bulbs (8 genera) to mildly rotten bulbs (36 genera), followed by a sharp decline in severely rotten bulbs (11 genera). Several bacterial genera, including *Lactobacillus*, *Novosphingobium*, *Sphingobium*, *Pluralibacter*, *Acetobacter*, *Gluconobacter* and *Pantoea*, emerged exclusively in rotten bulbs and were absent in healthy tissues, indicating their association with the onion bulb rot. The microbiome of SRB was marked by an overwhelming dominance of *Lactobacillus* (33.2% in SRB-CT, 16.9% in SRB-NT, 10.8% in SRB-OS), *Acetobacter* (16.1% in SRB-CT, 15.6% in SRB-NT, 7.0% in SRB-OS), *Carnimonas* (57.0% in SRB-NT), and *Gluconobacter* (14.5% in SRB-OS). Fungal communities exhibited a similar successional pattern: healthy bulbs showed negligible fungal presence except in the neck tissue (HB-NT), whereas mildly rotten bulbs showed a sharp increase in fungal diversity dominated by *Meyerozyma* (21.7%), *Blastobotrys* (13.3%), and *Penicillium* (7.0%). In severely rotten bulbs, fungal diversity declined, with *Pichia* (48.3%) and *Kazachstania* (8.6%) becoming dominant. Differential abundance analysis using edgeR identified six bacterial genera (*Lactobacillus*, *Novosphingobium*, *Acetobacter*, *Pluralibacter*, *Carnimonas*, and *Dysgonomonas*) and two fungal genera (*Pichia* and *Kazachstania*) that exhibited significant stage-dependent shifts during bulb rot progression. Alpha- and beta-diversity analyses revealed strong tissue-specific structuring of fungal communities, identifying the neck region as the primary fungal succession zone. Overall, this study elucidates the ecological restructuring of bacterial and fungal communities during onion bulb deterioration, and would pave the way for devising microbiome-informed interventions to reduce postharvest losses in onions.

## 1 Introduction

Onion (*Allium cepa* L.), one of the most widely cultivated and consumed vegetables, is an indispensable culinary ingredient across cultures worldwide. Onion is widely consumed both raw, such as in salads, and as a key component in diverse cuisines due to its characteristic flavor, aroma, and taste (Jin et al., 2024). Although onions are a relatively hardy bulb crop with a longer shelf life than many fresh vegetables, they are prone to considerable postharvest losses primarily due to microbial spoilage during storage and marketing. Existing reports suggest that postharvest losses in onion can range from 20 to 40% depending on storage conditions, cultivar., and geographical region, contributing to significant economic losses and food insecurity (Suravi et al., 2024). Bacterial and fungal infections are major contributors to onion bulb spoilage, intensifying rotting in mechanically damaged bulbs during storage and transport and causing up to 30% of total post-harvest losses (Sharma, 2023). During storage and transport, the onion bulbs positioned in lower layers of storage heaps are subjected to mechanical pressure from the overlying bulbs, resulting in bruising, cracking, or crushing of the bulb tissues, thereby predisposing such bulbs to microbial invasion and accelerated decay. Likewise, storage of onion bulbs in regions with high temperature and elevated relative humidity, such as tropical areas, not only accelerates bulb respiration but also creates favorable conditions for microbial growth, ultimately reducing bulb quality and shortening storage life (Kleman, 2023). Furthermore, the high moisture content and abundant simple sugars present in onion bulbs provides conducive microenvironment for the growth of a wide range of pathogens that contribute significantly to postharvest decay, incurring storage losses (Kiran et al., 2024). The improper curing and drying of onion bulbs prior to storage renders the bulb, neck, and outer scales/skins highly vulnerable to fungal and bacterial pathogens (Schroeder and Du Toit, 2010). Therefore, post-harvest spoilage of onion bulbs remains a major challenge for onion growers, traders, researchers, and stakeholders involved in the onion supply chain. Improper curing and drying of onion bulbs prior to storage renders the bulb, neck, and outer scales/skins highly vulnerable to fungal and bacterial pathogens.

Culture-dependent investigations of onion bulb pathogens have revealed a wide range of fungal pathogens that include different species of *Aspergillus, Penicillium, Fusarium, Botrytis, Rhizopus,* and *Colletotrichum*, as well as bacterial pathogens such as *Pseudomonas, Lactobacillus,* and *Erwinia*. These microorganisms are well recognized for their role in causing substantial post-harvest losses of onions during storage. *Fusarium* species are the most frequently reported, with *Fusarium oxysporum* f. sp. *cepae* being the causal agent of basal rot, a disease that can persist both in the field and during storage (Diabankana et al., 2024). *Aspergillus niger* is another major fungal pathogen responsible for black rot and is among the most frequently isolated fungi from infected onion bulb tissues (Alkasim et al., 2025). In addition, other storage fungi such as *Penicillium* spp. have been implicated in soft rot development in onion bulbs (Duduk et al., 2017). Bacterial pathogens such as *Burkholderia* spp. are known to cause sour skin disease and bulb rot in onion, leading to significant postharvest losses during storage and marketing (Paudel et al., 2024). Some other bacterial pathogens known to cause post-harvest onion spoilage include *Pantoea* spp. (De Armas et al., 2022)*, Pectobacterium carotovorum* (Zhang et al., 2023), *Dickeya* spp. (Ma et al., 2020)*, Rouxiella badensis* (Zhao et al., 2022) and *Pseudomonas* spp. (Khanal et al., 2022). Traditionally, the studies on these pathogens have relied on culture-based isolation, pathogenicity tests, and targeted molecular identification to establish their role in onion spoilage. While these approaches identify key pathogens like *Fusarium*, *Aspergillus*, and *Burkholderia*, they often overlook the broader microbial communities and potential synergistic interactions that drive disease progression. This underscores the need for microbiome-based studies to unravel complex community interactions beyond individual pathogens.

Microbial community–based investigations of onion spoilage are of considerable importance, as internal plant tissues naturally harbor many uncultured endophytic microorganisms that typically exist as symbionts or commensals but can transition to pathogenic forms under specific host-related physiological changes or environmental stresses (Kusstatscher et al., 2020). Therefore, a microbiome perspective highlights the complexity of microbial succession and functional shifts that occur during onion storage and subsequent rotting. Recent advances in microbiome research have revealed that both bacterial and fungal members play crucial roles in determining the progression of spoilage, often through enzymatic degradation of host tissues, competition, or synergistic interactions with other microbes (Kuruppu et al., 2024). Therefore, devising a rational biocontrol strategy for post-harvest bulb storage requires the identification of key microbial players causing bulb spoilage. A deeper understanding of bulb microbiome not only helps to identify the microbial drivers of bulb spoilage, but also highlights the role of opportunistic and functionally dominant taxa which may or may not be well-known pathogens but still crucial in bulb rotting.

While microbiome-based approaches have been applied to many perishable fruits and vegetables like tomato (Ottesen et al., 2013), apple (Shen et al., 2018), grapes (Mezzasalma et al., 2018), oranges (Wang et al., 2018), sugar beets (Kusstatscher et al., 2019) and kiwifruit (Wu et al., 2019), the microbiome associated with onion bulbs, particularly the shifts between healthy and diseased states and tissue-specific microbiome, remains poorly understood. A major contributor to onion spoilage is the dynamic interaction between the bulb tissue and associated microbial communities. Increasing evidence suggests that rot symptoms are often associated with multiple microbial taxa rather than a single pathogen, and that interactions among these microbes can accelerate tissue maceration. Culture-independent amplicon sequencing studies on fruit rot, such as grapevine, have revealed that decaying tissues harbor complex bacterial and fungal communities comprising dozens of genera like *Pichia*, *Zygosaccharomyces*, *Gluconobacter* and *Acetobacter*, while the traditionally recognized primary pathogens constitute only a subset of the rot-associated microbiome (Brischetto et al., 2024). Therefore, the development of novel and robust disease-control strategies relies on understanding the dynamics of disease emergence on the level of plant-microbiome interaction (Wisniewski and Droby, 2019). This necessitates understanding the microbial interaction of the onion spoilage to devise rather more effective ways to combat spoilage losses based on the microbiome-assisted insights of onion spoilage. To address this gap, the present study investigates bacterial and fungal microbiome dynamics in healthy versus diseased onion bulbs using 16S and ITS amplicon sequencing. By identifying taxonomic differences and key microbial signatures associated with bulb spoilage, this study aims to provide baseline knowledge for developing microbiome-informed strategies to mitigate postharvest onion losses.

## 2 Materials and methods

### 2.1 Bulb selection, categorization, and tissue sampling

Uniform size onion bulb samples of the onion cultivar ‘*Bhima kiran*’ were collected in Oct 2024 from the harvest lot of *rabi*-2024 stored in open ventilated conventional storage structures (Location: 18.84414° N, 73.88519° E) of ICAR-Directorate of Onion and Garlic Research, Pune, India. The storage environment was unregulated, simulating traditional farmer-level storage practices, which typically expose the bulbs to fluctuating ambient temperature and relative humidity. No artificial temperature or humidity control was applied during storage. The temperature and humidity data of the storage months (April-Oct 2024) for the storage location were recorded at on-site automated weather monitoring station installed at ICAR-DOGR approximately 300 m from the onion bulb storage facility (Supplementary Table S1).

Based on visual inspection, five onion bulbs of uniform size were selected for each of three categories: healthy bulbs (HB), mildly rotten bulbs (MRB), and severely rotten bulbs (SRB). The bulbs which did not show any visible sign of damage or decay with complete integrity of outer skin and unexposed neck region, absence of any unusual odor, and no tissue exudation, were selected as healthy bulbs (HB). Since the bulbs were stored in plastic crates, care was taken not to select any bulb having rotted bulb in its vicinity. The bulbs exhibiting early-stage visible symptoms of progressive decay, limited softening of the outer scales, slight discoloration, and the development of localized lesions and mild foul odor were selected as mildly rotten bulbs (MRB). The onion bulbs showing advanced visible symptoms of decay with extensive softening of internal tissues, tissue liquefaction, and the presence of a severe foul odour were selected as severely rotten bulbs (SRB) for the microbiome studies. Detailed visible characters used for bulb category selection are provided in Supplementary Table S2. The selected bulb samples of healthy bulbs (HB), mildly rotten bulbs (MRB), and severely rotten bulbs (SRB) were sampled in a sterile, autoclaved bags and immediately transported to the laboratory for tissue sampling under aseptic conditions.

One representative bulb from each category (HB, MRB, and SRB) was aseptically dissected under a laminar airflow hood using sterile surgical blades to collect three distinct tissues: neck tissue (NT), outer scale tissue (OS), and central tissue (CT), each of approximately 10 cm^2^. This resulted in a total of nine distinct sample types representing 3 neck tissue (HB-NT, MRB-NT, SRB-NT), 3 outer scale tissues (HB-OS, MRB- OS, SRB- OS) and 3 central tissues (HB-CT, MRB- CT, SRB-CT). All tissue samples were immediately stored at −80 °C until DNA extraction.

### 2.2 DNA extraction, NGS library preparation, and amplicon sequencing

Metagenomic DNA was extracted from different bulb tissues using the XpressDNA Plant kit (MagGenome) as per the manufacturer’s protocol (Das et al., 2023) with minor adaptation for bulb tissue. Briefly, approximately 100 mg of frozen bulb tissue was finely ground in liquid nitrogen and the powdered tissue was transferred to a 2 mL microcentrifuge and was subjected to chemical lysis using proprietary plant lysis buffers (200 μL of buffer A and 650 μL buffer B). For soft tissues like bulb, 85 μL of buffer C was used. DNA was isolated using magnetic nanoparticle-based binding, followed by sequential wash steps to remove inhibitors. The purified DNA was eluted in 50 μL elution buffer. The quality of the extracted DNA samples was analysed using NanoDrop™ spectrophotometer (Thermo Fisher Scientific, USA) by determining the A260/280 absorbance ratio. The amplicon libraries were prepared from the extracted DNA using primer pair 341F (5′- CCTAYGGGRBGCASCAG-3′) and 806R (5-GGACTACNNGGGTATCTAAT-3′) targeting the V3–V4 hypervariable regions of the 16S rRNA gene (He et al., 2021). For fungal community analysis, the internal transcribed spacer (ITS) region was amplified using the primer pair ITS1 (5′-TCCGTAGGTGAACCTGCGG-3′) and ITS4 (5′-TCCTCCGCTTATTGATATGC-3′) (Toju et al., 2012). The thermal cycling conditions consisted of an initial denaturation at 95 °C for 3 min, followed by 25 cycles of denaturation at 95 °C for 30 s, annealing at 55 °C for 30 s, and extension at 72 °C for 30 s, with a final elongation step at 72 °C for 5 min. The amplified products were purified using AMPure XP beads (Beckman Coulter, USA) and Illumina sequencing adapters were added using the Nextera XT Index Kit (Illumina, USA) as per the manufacturer’s protocol. Library quality and size distribution were evaluated using the Agilent 4,200 TapeStation system with the D1000 ScreenTape kit (Agilent Technologies, USA). Libraries exhibiting a corresponding peak at the expected fragment size for V3-V4 and ITS were quantified using a Qubit fluorometer (Thermo Fisher Scientific, USA) and normalized to equimolar concentrations. The pooled libraries were subjected to paired-end sequencing (2 × 300 bp) on the Illumina MiSeq platform (Illumina, USA) at Eurofins Genomics, Bengaluru, India, Pvt. Ltd.

### 2.3 Bioinformatic and statistical analysis

The raw Illumina paired end reads generated in this study were checked for their quality using the MultiQC (Ewels et al., 2016). The poor-quality reads were trimmed using the Trimmomatic (Bolger et al., 2014), and only the good quality reads (Q > 30) were retained for downstream analysis for generating the microbiome profiles of each sample employing the QIIME2 (version QIIME2-2022.2) (Bolyen et al., 2019). The QIIME2 analysis included denoising of the reads by removing non-biological sequences using DADA2 (Callahan et al., 2016), followed by merging of R1 and R2 reads to generate a table of amplicon sequence variants (ASVs) along with a corresponding set of dereplicated representative sequences. Taxonomic classification of the representative ASVs was performed using the machine-learning classifier approach implemented in QIIME 2 (feature-classifier classify-sklearn) for bacterial sequences. A naïve Bayes classifier was trained on the SILVA 16S rRNA reference database, restricted to the V3–V4 region corresponding to the sequencing primers used in this study. The SILVA reference sequences were extracted with the same primer pair using the extract-reads method to ensure region-specific training. The classifier was then trained on these trimmed reference sequences and the corresponding SILVA taxonomy file. ASVs were assigned taxonomy using the trained classifier with default parameters (confidence threshold = 0.7).

For fungal community analysis, the raw ITS amplicon reads were processed using the ITSxpress to extract the fungal ITS region with improved precision (Einarsson and Rivers, 2024). Quality-filtered ITS reads were subsequently subjected to taxonomic assignment using a BLAST-based approach (q2-feature-classifier classify-consensus-blast) against the UNITE fungal ITS reference database (Abarenkov et al., 2024). The resulting taxonomic tables were used for downstream community composition and diversity analyses. Since the sequencing depth varied among samples, the feature table was rarefied to the sequencing depth of the sample with the lowest number of reads to retain all samples for downstream comparative analyses of alpha- and beta-diversity analyses, whereas taxonomic composition was assessed using relative abundance–normalized, non-rarefied data to avoid the loss of low-abundance taxa. Host-derived chloroplast- and mitochondrial-affiliated sequences were not removed to avoid excessive loss of reads from plant tissue samples, where host-derived organellar DNA is abundant and removal could substantially reduce sequencing depth and therefore all samples were processed consistently, and comparative analyses were therefore not biased by differential filtering across sample types. The taxonomy barplot, differential abundance analysis, alpha and beta diversity analysis (NMDS, Non-metric Multidimensional Scaling ordination plots) were generated using various related plugins implemented in QIIME2 and functionality implemented in Microbiomeanalyst 2.0 (Lu et al., 2023).

## 3 Results

### 3.1 Bacterial community structure in healthy, mildly rotten, and severely rotten onion bulbs

The bacterial community structure was inferred from a total of 854,720 Illumina 16S rRNA V3–V4 reads generated across nine onion bulb samples (Supplementary Table S3), representing the neck tissue (NT), central tissue (CT), and outer scale (OS) from healthy bulbs (HB), mildly rotten bulbs (MRB), and severely rotten bulbs (SRB). After quality filtering and denoising, 466,058 high-quality sequences were retained, yielding 1,368 distinct ASVs across nine samples (mean ASVs = 207) (Supplementary File 1). A substantial fraction of the ASVs (526) were taxonomically identified as mitochondrial or chloroplast origin, and this proportion was markedly higher in healthy bulb (HB) samples than in mildly rotten (MRB) and severely rotten (SRB) bulbs, indicating a comparatively lower bacterial load in healthy bulb tissues. The taxonomic assignments of bacterial features resulted in 85 OTUs across nine tissue samples (Supplementary File 2).

#### 3.1.1 Phylum-level shifts in the bacterial community during onion bulb rot

**Figure 1:**
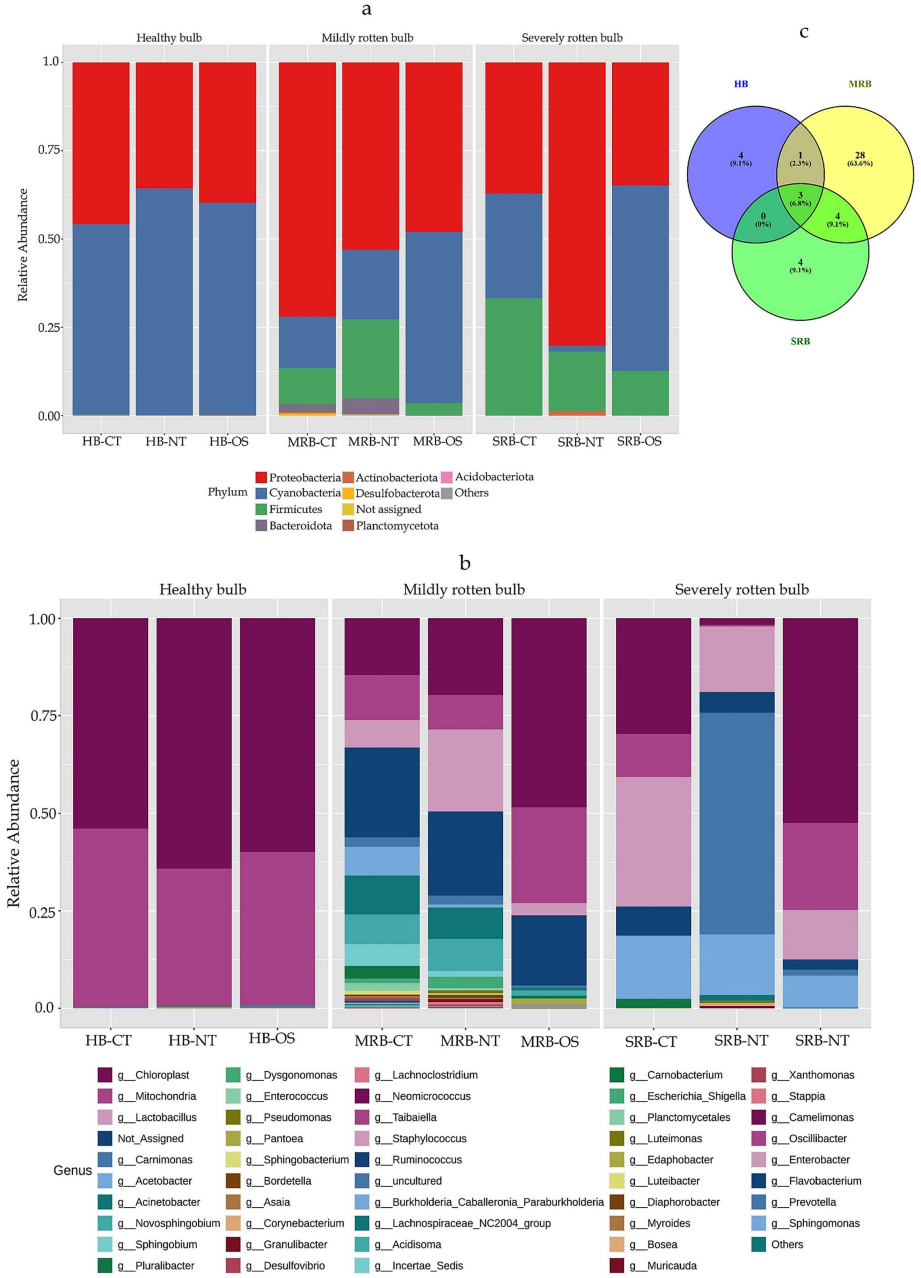
Phylum-level **(a)** and genus-level **(b)** relative abundance of bacterial communities across healthy and rotten bulb tissues with varying rot severity. Stacked bar plots show the relative abundance (%) of dominant bacterial genera detected in nine onion bulb tissue samples representing healthy bulbs (HB), mildly rotten bulbs (MRB), and severely rotten bulbs (SRB). The relative abundances have been calculated by including host derived sequences. Each bars represent sample-wise relative abundances calculated from total reads without removing host-derived and unassigned sequences. **(c)** Venn diagram showing the number of shared and unique taxa among HB, MRB, and SRB. Overlapping areas indicate taxa common between groups, while non-overlapping areas represent taxa unique to each group.

Although healthy bulbs (HB) exhibited far fewer OTUs (15) than MRB (68) and SRB (22) at similar sequencing depth, four bacterial phyla, Proteobacteria, Firmicutes, Bacteroidota, and Planctomycetota were nonetheless detected in HB tissues. Analysis of phylum-level composition across the three bulb conditions showed distinct shifts in bacterial diversity (Figure 1a). While healthy bulbs (HB) harbored OTUs from only four phyla, the mildly rotten bulbs (MRB) displayed considerably higher richness, encompassing seven phyla: Acidobacteriota, Actinobacteriota, Bacteroidota, Desulfobacterota, Firmicutes, Planctomycetota, and Proteobacteria. In contrast, severely rotten bulbs (SRB) contained only three phyla (*Proteobacteria*, *Firmicutes*, and *Actinobacteriota*). Thus, microbial diversity increased during the early stages of rot (MRB) and declined in advanced rot (SRB), indicating a shift from a low-diversity healthy bulb microbiome to a more complex community during initial decay, followed by dominance of fewer phyla in severely degraded tissues.

#### 3.1.2 Genus-level changes in bacterial communities with progressive bulb rotting

Genus-level comparison of HB, MRB and SRB microbiome revealed a pronounced shifts in community composition with the progression of bulb rot. The bacterial microbiome profiles of healthy bulb (HB) tissues were characterized by a strong dominance of host-origin sequences (89%) cumulatively in all three tissues, while the associated bacterial genera occurred at low relative abundance and displayed tissue-specific distribution patterns. The neck tissue (HB-NT) harbored low abundances of *Acinetobacter* (0.26%), *Luteimonas* (0.17%), *Staphylococcus* (0.16%), and *Pseudomonas* (0.14%). In contrast, the outer scale tissue (HB-OS) contained *Carnimonas* (0.26%), *Acinetobacter* (0.17%), *Muricauda* (0.12%), and *Stappia* (0.10%) (Figure 1b). The bacterial genera *Carnobacterium* (0.23%), *Enterobacter* (0.08%), and *Prevotella* (0.07%) were detected exclusively in the central tissue (HB-CT). The presence of limited taxa in very low abundance indicates a relatively simple and tightly regulated bacterial community in HB, which is commonly observed in healthy, intact plant tissues. In contrast, mildly rotten bulbs (MRB) exhibited a substantial expansion in bacterial diversity, comprising 36 genera (Figure 1c) with tissue-specific differential mictobiome profiles. The MRB-CT predominantly included *Acinetobacter* (10.7%), *Novosphingobium* (8.2%), *Lactobacillus* (7.6%), *Sphingobium* (6.0%), and *Pluralibacter* (3.5%) whereas the neck tissue (MRB-NT) exhibited a distinct profile, characterized by a strong enrichment of *Lactobacillus* (21.4%), followed by *Novosphingobium* (8.4%), *Acinetobacter* (8.2%), *Granulibacter* (0.77%), *Diaphorobacter* (1.19%), and *Lachnoclostridium* (0.66%). In the outer scale tissue (MRB-OS), bacterial abundances were comparatively lower but included *Lactobacillus* (3.1%), *Pantoea* (1.5%), *Novosphingobium* (1.3%), *Pluralibacter* (0.69%), and *Burkholderia–Caballeronia–Paraburkholderia* (0.57%).

The bacterial microbiome profiles of severely rotten onion bulbs (SRB) revealed a pronounced decline in community complexity, with only 11 genera detected and dominance of a few taxa, suggesting strong selective pressure imposed by advanced tissue decay. The central tissue (SRB-CT) was characterized by a strong enrichment of *Lactobacillus* (33.2%) and *Acetobacter* (16.1%), whereas neck tissue (SRB-NT) was dominated by *Carnimonas* (57.0%) followed by *Lactobacillus* (16.9%) and *Acetobacter* (15.6%). In contrast, the outer scale tissue exhibited a more heterogeneous bacterial composition, dominated by *Lactobacillus* (10.8%), *Acetobacter* (7.0%), and *Gluconobacter* (14.5%), with lower abundances of *Carnimonas* (1.3%).

The analysis of shared and unique genera of each sample types showed that healthy bulbs (HB) harbored a limited bacterial community comprising eight genera, whereas mildly rotten bulbs (MRB) showed the highest richness with 36 genera, and severely rotten bulbs (SRB) contained 11 genera. Three genera namely *Acinetobacter*, *Pseudomonas*, and *Carnimonas* were detected across all three bulb conditions; however, their relative abundance was notably higher in MRB and SRB bulbs. Healthy bulb (HB) harbored four unique genera (*Carnobacterium*, *Luteimonas, Enterobacter,* and *Prevotella*), MRB contained the highest number of unique genera (28), while SRB showed five unique genera (*Gluconobacter, Corynebacterium, Neomicrococcus, Providencia,* and *Escherichia–Shigella*) (Figure 1c).

### 3.2 Fungal diversity patterns in healthy vs. rotten onion bulbs

A total of 1,107,897 Illumina ITS reads generated from nine bulb tissue samples were used to infer the fungal community structure (Supplementary Table S4) resulting in 195 distinct ASVs (Supplementary File 3) and 53 OTUs (Supplementary File 4) across nine bulb tissue samples.

#### 3.2.1 Shifts in fungal communities with bulb rotting

**Figure 2:**
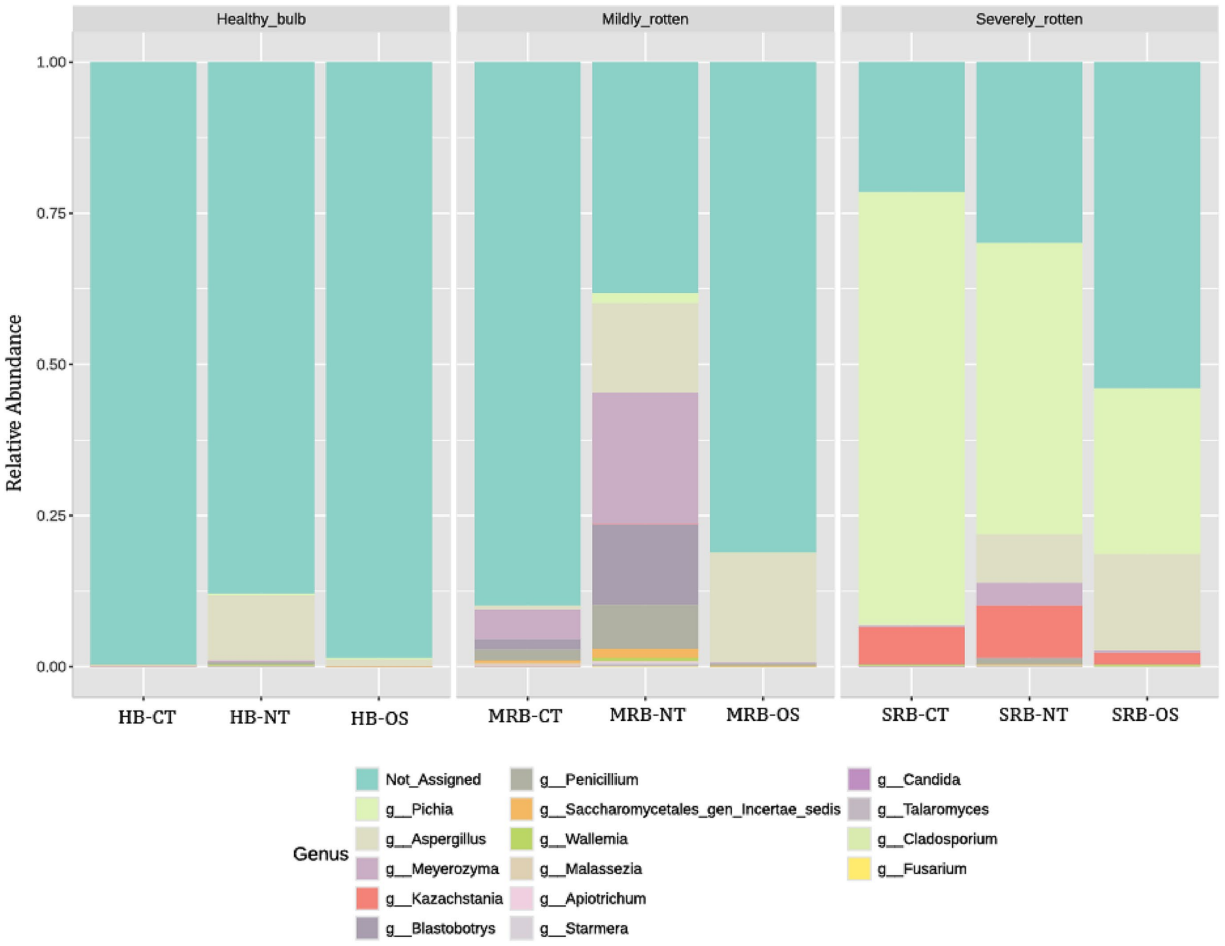
Genus-level relative abundance of fungal communities associated with onion bulb tissues exhibiting different rot severities. Stacked bar plots depict the relative abundance (%) of dominant fungal genera detected across nine onion bulb tissue samples, comprising healthy bulbs (HB), mildly rotten bulbs (MRB), and severely rotten bulbs (SRB). Relative abundances were calculated on a sample-wise basis using total assigned reads, including host-derived sequences, without prior host read removal.

The fungal colonization in healthy bulbs was minimal, although the neck tissue (HB-NT) displayed comparatively higher colonization, with *Aspergillus* accounting for approximately 10% of the total reads. In mildly rotten bulbs, the neck tissue (MRB-NT) showed a clear shift marked by the emergence of new fungal genera viz. *Meyerozyma* (21.7%), *Blastobotrys* (13.3%), and *Penicillium* (7%) as dominant members of the community (Figure 2). Notably, these genera were completely absent in healthy neck tissue (HB-NT). Alongside the appearance of these new taxa, *Aspergillus* also exhibited a modest increase in MRB-NT, rising from 10% in healthy bulbs (HB-NT) to about 13% in MRB-NT. As rot progressed to the severe stage, the neck tissue (SRB-NT) exhibited pronounced mycobiome restructuring, with *Pichia* dominating (48.3%) along with *Kazachstania* (8.6%), *Aspergillus* (8.0%), and *Meyerozyma* (3.7%).

While mycobiome shifts were evident in the outer scales and central tissues, these changes were less pronounced than in the neck tissue, with mildly rotten outer scales (MRB-OS) showing increased *Aspergillus* abundance (18.2%). In contrast, in the severely rotten stage (SRB-OS), *Pichia* (27.4%) emerged as the most dominant fungal genus in later stages of rot progression (Figure 2). The central tissue of the healthy onion bulb showed minimal fungal colonization, as reflected by the high proportion of unassigned sequences (99%). Even in the mildly rotten stage (MRB-CT), the fungal load remained comparatively low, with 90% of sequences still unassigned. Among the identifiable taxa, *Meyerozyma* (4%), *Penicillium* (1%), and *Blastobotrys* (1%) were detected, indicating the early and limited infiltration of only a few opportunistic fungi into the central tissues during the initial stages of rot. However, the severe rotting of the bulb was accompanied by the sharp increase in *Pichia* (71%) and *Kazachstania* (6%) even in the central part of the onion bulb (SRB-CT).

### 3.3 Alpha and beta-diversity patterns across bulb conditions and tissues

Alpha diversity comparisons for both bacterial and fungal communities were conducted on datasets rarefied to the minimum sequencing depth observed across samples. The rarefaction curves reached saturation at the selected rarefaction depth, confirming that the chosen sequencing depth was sufficient for robust alpha diversity comparisons (Supplementary Figure S1). Alpha diversity analysis revealed clear differences in bacterial and fungal community richness and evenness across bulb tissue and bulb stages. The highest bacterial richness was observed in MRB-CT and MRB-NT with Chao estimates of 42 and 41, respectively (Table 1), along with comparatively high Shannon (2.88 and 2.65) and Simpson indices (0.92 and 0.90), indicating a diverse and relatively even community. In contrast, the outer-scale tissue of MRB (MRB-OS) had lower bacterial richness (Chao = 20) and diversity (Shannon = 1.75; Simpson = 0.76), suggesting dominance of fewer bacterial taxa in the outer scales of MRB. The SRB samples showed overall lower richness and diversity, with a Chao index value of 12 and moderate Shannon (1.92) and Simpson (0.83) indices. The neck tissue (SRB-NT) displayed slightly higher richness (Chao = 17) but the lowest diversity within SRB samples (Shannon = 1.50; Simpson = 0.63), indicating increased dominance by selected bacterial taxa.

**Table 1:** Alpha diversity indices of bacterial and fungal communities associated with healthy (HB), mildly rotten (MRB) and severely rotten (SRB) bulb tissues.

Sample	Diversity indices (bacterial community)				Diversity indices (fungal community)			
	Chao	Shannon	Simpson	Fisher	Chao	Shannon	Simpson	Fisher
MRB-CT	42	2.88	0.92	4.74	18	0.52	0.20	1.71
MRB-NT	41	2.65	0.90	4.61	28.5	1.74	0.77	2.79
MRB-OS	20	1.75	0.76	2.06	19	0.54	0.31	1.81
SRB-CT	12	1.92	0.83	1.17	21	0.81	0.44	2.02
SRB-NT	17	1.50	0.63	1.72	23.5	1.42	0.67	2.13
SRB-OS	12	1.87	0.81	1.17	24	1.13	0.61	2.35
HB-CT	6	1.02	0.61	0.54	17	0.04	0.01	1.60
HB-NT	7	1.11	0.65	0.64	18	0.53	0.25	1.71
HB-OS	8	1.13	0.66	0.75	18.5	0.10	0.03	1.71

Almost similar trends were observed for alpha diversity indices for fungal communities. The MRB samples, particularly the neck tissue (MRB-NT), exhibited the highest richness and diversity, with a Chao estimate of 28.5, Shannon index of 1.74, Simpson index of 0.77, and the highest Fisher’s alpha (2.79), suggesting a comparatively diverse and evenly distributed fungal community. In contrast, the control (MRB-CT) and outer-scale tissue (MRB-OS) showed lower diversity and evenness, as reflected by low Shannon (0.52 and 0.54) and Simpson values (0.20 and 0.31), indicating dominance of a few taxa despite comparable richness. Overall, MRB samples harbored the most diverse bacterial and fungal communities, SRB samples showed intermediate diversity, and HB samples were characterized by low richness, highlighting distinct mycobiome structures across sample categories and rotting stages.

**Figure 3:**
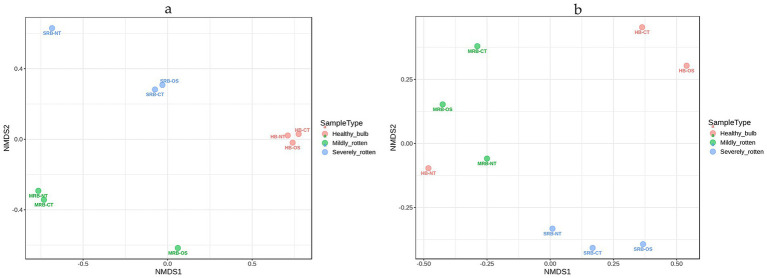
Non-metric multidimensional scaling (NMDS) ordination plots showing differences in bacterial community **(a)** and fungal community **(b)** composition among healthy (HB), mildly rotten (MRB), and severely rotten (SRB) onion bulbs across neck tissue (NT), outer scale (OS), and central tissue (CT). Each point represents an individual sample, with colors indicating disease status. Spatial separation of samples reflects dissimilarities in community structure. The Bray–Curtis dissimilarity matrix was used for ordination to visualize differences among samples.

NMDS ordination plots based on bacterial community showed a clear 55% of the variation in microbial community composition according to bulb health status (Figure 3a). The samples clustered distinctly according to rot stage, with healthy bulbs tissues (HB) forming a compact cluster, indicative of more comparable microbial composition of healthy bulbs irrespective of tissue type. The ordination plots revealed a more pronounced effect of rot stage than tissue type on microbiome profiles. In addition to rot-stage separation, bacterial beta diversity patterns also highlighted tissue-specific structuring, particularly in MRB and SRB samples, where neck, outer scale, and central tissues occupied distinct ordination spaces. Notably, neck tissues of MRB and SRB diverged more from healthy profiles, supporting their role as a primary site of microbial entry and early colonization during bulb decay.

The NMDS ordination based on the fungal community showed a different pattern, in which severely rotten bulb samples (SRB) clustered distinctly and were clearly separated along the NMDS axes from both HB and MRB samples, demonstrating a pronounced restructuring of the fungal microbiome at advanced stages of decay. The tighter grouping of SRB samples suggests reduced community complexity and dominance of a limited number of decay-adapted fungal taxa under advanced rot conditions. The mildly rotten bulbs tissue overlapped with HB-NT (healthy bulb neck tissue) samples reflecting an early or latent fungal colonization in the neck region, which may occur even in visually healthy bulbs (Figure 3b).

### 3.4 Differential abundance of bacterial and fungal taxa in healthy and rotten bulbs

**Figure 4:**
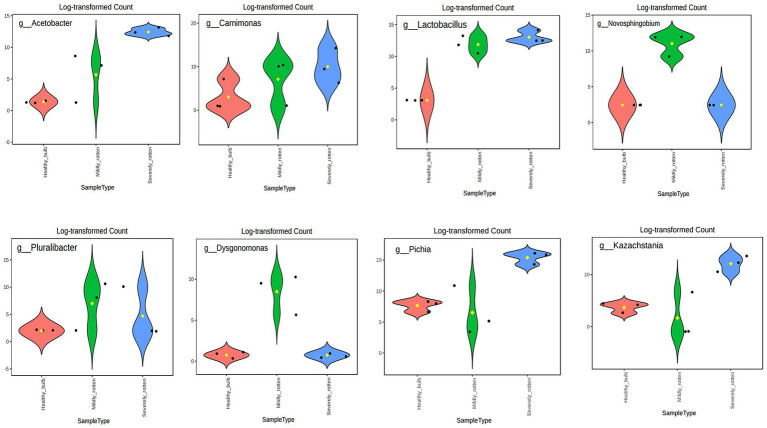
Differential abundance of bacterial and fungal genera across onion bulb health stages. Violin plots show the distribution of normalized and log_2_-transformed counts for differentially abundant bacterial and fungal genera identified using edgeR across healthy bulbs (HB), mildly rotten bulbs (MRB), and severely rotten bulbs (SRB).

Differentially abundant bacterial and fungal taxa were identified using edgeR across healthy, mildly rotten, and severely rotten bulb samples. The edgeR identified 6 bacterial genera (*Lactobacillus*, *Novosphingobium*, *Acetobacter*, *Pluralibacter*, *Carnimonas, Dysgonomonas*) and 2 fungal genera (*Pichia* and *Kazachstania*) showing differential abundance across bulb stages (Figure 4). The genus *Lactobacillus* showed significantly higher abundance in MRB (log2FC = 6.83, FDR = 0.001) and SRB (log2FC = 12.65, FDR = 0.001) compared to HB. Similarly, the genera *Acetobacter* (log2FC = 11.9, FDR = 0.001) and *Carnimonas* (log2FC = 10.15, FDR = 0.001) showed significantly higher abundance in SRB compared to MRB. The abundance of the genus *Dysgonomonas* decreased in SRB compared to MRB (log2FC = −2.71, FDR = 0.04), whereas the fungal genera *Pichia* (log2FC = 10.8, FDR = 0.001) and *Kazachstania* (log2FC = 11.3, FDR = 0.001) showed significantly higher abundance in SRB relative to MRB.

## 4 Discussion

In view of the significant postharvest losses encountered during onion bulb storage, understanding the compositional dynamics of microbial communities associated with the rot process is essential for developing effective management strategies. Although several fungal and bacterial species like *Botrytis allii*, *Fusarium oxysporum*, *Aspergillus niger*, and *Burkholderia cepacia* are known as causal agents of onion bulb decay, the accumulating evidence suggests that rot development is better explained as a microbiome-driven process (pathobiome), arising from dynamic microbial interactions rather than the action of a single pathogenic organism (Droby et al., 2022). This study documented the transitions in both bacterial and fungal community structures across healthy, mildly rotten, and severely rotten onion bulbs, clearly demonstrating that bulb spoilage is a dynamic and microbiome-driven successional process shaped by microbial interactions and host tissue degradation. Overall, a total of 85 bacterial and 53 fungal OTUs were detected across bulb samples representing different stages of rot severity, including healthy, mildly affected, and severely rotten tissues. The presence of 85 bacterial and 53 fungal OTUs in bulb tissues indicates a moderate taxonomic diversity, especially for an internal plant tissue such as an onion bulb, which is considered a selective niche for microbial colonization. In line with this observation, an earlier study by Yurgel et al. reported the presence of 149 bacterial and 40 fungal OTUs across healthy and diseased onion, highlighting taxonomically diverse communities (Yurgel et al., 2018). Even though the healthy onion bulbs exhibited very low microbial colonization, with approximately 89% of the total sequences corresponded to host-derived chloroplast/mitochondrial DNA. This dominance of host sequences indicates that the internal tissues of healthy bulbs harbor a substantially lower microbial load compared to tissues from rotten onion bulbs. The dominance of host-derived sequences in healthy onion bulb tissues is a well-documented phenomenon, with studies such as Yurgel et al. reporting that ~95% of sequences recovered from healthy bulbs were of host chloroplast origin in comparative microbiome analyses of healthy and diseased tissues (Yurgel et al., 2018). Conversely, some amplicon sequencing–based studies have reported no detectable bacterial ASVs altogether in asymptomatic healthy bulbs; nevertheless, this observation could be a technical limitation arising from low sequencing coverage, rather than evidence for the absence of bacterial endophytes (Liakos et al., 2025). Since, in the present study, the rarefaction analysis revealed the saturation with no further increase in species richness with sequencing depth in healthy bulbs (Supplementary Figure S1), it is likely that the observed patterns reflected good capture of the microbial diversity present in the healthy onion bulbs. Despite the lower microbial abundance in healthy bulbs, OTUs representing four phyla (Proteobacteria, Firmicutes, Bacteroidota, and Planctomycetota) and eight bacterial genera were detected, indicating that although microbial presence was low, the healthy bulb environment still harbored a diverse bacterial community. Since onions are widely consumed raw in the form of salads, the insights in to microbiome of healthy bulbs may have health implications. The presence of bacterial sequences representing genera like *Prevotella*, especially in central tissues of healthy bulbs, is noteworthy, given that members of this genus are well-recognized beneficial inhabitants of the human gut microbiome (Tett et al., 2021). These findings suggest that raw onion consumption may serve as a source of beneficial gut-associated bacteria such as *Prevotella*; however, this remains a preliminary inference based on the prevalence of *Prevotella* sequences in healthy bulbs, in light of existing studies describing diet-mediated acquisition of the human gut microbiome (Wicaksono et al., 2023), and therefore requires further validation through strain-level, culture-based studies.

Our analysis of the bacterial community revealed that the bacterial genera like *Acinetobacter*, *Pseudomonas*, and *Carnimonas* were detected in both healthy and rotten onion bulbs, suggesting that these are inhabitant taxa capable of surviving in intact tissues as well as proliferating under conditions of tissue degradation. Although present at a very low abundance in healthy bulbs, their relative abundance increased markedly in mildly and severely rotten bulbs, indicating that taxa respond to changed biochemical environment of the bulb during the rotting process. Their increased abundance also supports the view that these bacteria may persist in a latent or quiescent state within healthy bulbs and proliferate as the bulb microenvironment becomes permissive during decay. The detection of these genera in healthy bulb tissues, and their increased abundance during rot progression, suggests that they may persist as low-abundance resident taxa and proliferate as the bulb microenvironment becomes permissive, raising the possibility of vertical acquisition as part of the native bulb microbiota (Pereira et al., 2023). The presence of *Acinetobacter* and *Pseudomonas* in the healthy onion bulbs and their increased abundance with onion bulb rot has been reported previously in uncultured survey of onion bulbs from Annapolis Valley, Canada (Yurgel et al., 2018). Moreover, several studies have reported the frequent occurrence of *Acinetobacter* in many fruits and vegetables as their native microflora (Karumathil et al., 2016; Ababneh et al., 2022). In contrast, other genera such as *Lactobacillus*, *Novosphingobium*, *Acetobacter*, *Sphingobium*, *Pluralibacter*, and *Pantoea* were observed only in rotten bulbs, suggesting that they represent opportunistic secondary invaders of the onion bulb during progressive bulb rotting. The sharp increase in abundance observed in MRB suggests that early tissue damage and partial loss of bulb integrity create permissive niches that allow colonization of these taxa possibly through horizontal acquisition as their appearance during storage points to their acquisition through air, handling surfaces, storage crates, and soil residues (Frank et al., 2017). The neck tissue appears to function as a primary entry point for microbial invasion, as evidenced by the pronounced enrichment of diverse bacterial genera in MRB-NT. The neck region is anatomically more exposed and physiologically vulnerable, particularly under unregulated storage conditions, making it susceptible to horizontal microbial ingression. Once established in the neck tissue, these microorganisms may progressively spread to the outer scales and central tissues, following nutrient gradients created by tissue softening and cell wall breakdown. The bacterial genera like *Lactobacillus* (Bonasera et al., 2017), *Pantoea* (Zhang et al., 2022) are well described pathogens of onion bulb, the role of *Novosphingobium*, *Sphingobium, Pluralibacter* and *Acetobacter* has not been previously described in onion bulb decay, and therefore these taxa may represent opportunistic or saprophytic colonizers that proliferate following the tissue damage or changes in bulb tissues during storage. The detection of these taxa may also reflect ecological succession within the bulb microenvironment, where shifts in oxygen availability, pH, and available carbon sources during rot selectively favor certain bacterial groups. Overall, these findings support a horizontal environmental acquisition of microbes where initial tissue damage facilitates microbial ingression from the environment, followed by niche-driven selection and dominance of decay-adapted taxa as the bulb rot progresses to advance stages (SRB). In a shotgun metagenomic study, metagenome-assembled genomes (MAGs) affiliated with *Acetobacter* were recovered from symptomatic, decaying onion bulbs (Liakos et al., 2025). However, the absence of onion pathogenicity-related genes in the metagenome-assembled genomes (MAGs) of *Acetobacter* suggests that they function as opportunistic colonizers rather than primary pathogens. Collectively, these findings suggest that the onset of onion bulb rotting is associated with a gradual shift from a restricted, latent microbial community to a more diverse, enriched opportunistic assemblage rather than the single dominant pathogen driven process. Microbial diversity analysis was used to assess the transition from a relatively low-diversity microbiome in healthy bulb (HB) tissue to a more diverse and complex community in mildly rotten bulbs (MRB), followed by a marked decline in microbial diversity in severely rotten bulbs (SRB). The loss of microbial diversity is often associated with disease development across plant, animal, and human systems (Trivedi et al., 2020). In plant systems, this relationship is best documented in soils and rhizospheres, where healthy or disease-suppressive soils consistently exhibit higher microbial diversity and greater network complexity than disease-conducive soils (Dundore-Arias et al., 2023). Mendes et al. (2011) demonstrated that suppression of *Rhizoctonia solani* relied on a diverse and interactive microbial consortium rather than a single antagonistic taxon, and that disruption of this diversity compromised disease resistance. Similarly, Berendsen et al. (2012) and Trivedi et al. (2020) emphasized that loss of microbial diversity reduces ecosystem resilience and increases susceptibility to pathogen invasion.

Beyond plant systems, a similar pattern is observed in animal and human hosts, particularly in the gut, where high microbial diversity is observed for healthy states and the loss of microbial diversity is to inflammatory and infectious diseases (Lozupone et al., 2012). However, the association between loss of microbial diversity and disease is not universal and is highly system-dependent. In internal plant tissues and endophytic systems, where tight host filtering operates, the healthy state is characterized by lower but stable microbial diversity. In such systems, disease state can be associated with increased microbial diversity due to relaxed host control and invasion by opportunistic or saprophytic microorganisms. Consequently, the endophytic microbial composition is strongly influenced by plant genotype, tissue type, host defense responses, and the local metabolite landscape that collectively regulate microbial entry and persistence. The lower microbial diversity in healthy onion bulb tissues can be attributed to effective host filtering that maintains a restricted yet stable microbial community. The transient increase in diversity in MRB tissues is attributed to the weakening host control and increased microbial ingression during early decay. The subsequent decline in diversity in SRB tissues reflects the dominance of a few decay-adapted taxa under advanced tissue degradation. In light of strong host filtering as a driver of low diversity in healthy plant tissues, the human gut presents an interesting contrast, where active host filtering coexists with high microbial diversity in healthy states and a loss of diversity during disease. In the gut, host filtering operates within an open, nutrient-rich ecosystem, where high microbial diversity provides functional redundancy, colonization resistance, and metabolic stability, supporting host health. Thus, the relationship between microbial diversity and host health is system-dependent, shaped by host physiology, tissue type, nutrient availability, and the ecological role of the microbiome.

A marked reduction in microbial diversity in SRB tissues accompanied by dominance of a few bacterial genera like *Lactobacillus*, *Acetobacter*, *Carnimonas*, and *Gluconobacter* was observed. The bacterial genera such as *Lactobacillus* and *Gluconobacter*, which are enriched in advanced rot stages, are primarily fermentative or oxidative bacteria. These taxa are known to produce organic acids (lactic acid, gluconic acid, acetic acid), leading to localized acidification of host tissues. Thus, these bacteria may indirectly accelerate rot progression by making the bulb microenvironment more permissive for decay rather than directly degrading plant polymers. This suggests that the advanced stages of bulb rotting are accompanied with collapse in microbial community complexity. This shift is due to the changed microenvironment of the bulb as a result of loss of bulb tissue integrity and consequent enrichment of fermentable sugars, organic acids, creating acidic microenvironments favoring proliferation of acid-tolerant, fermentative, and oxidative bacteria (Parra-Aguilar et al., 2025). These observations suggest that microbial dysbiosis in rotting bulbs is largely a consequence of tissue decay rather than its sole cause. Our findings in the context of onion bulb rot, suggest that the tissue degradation likely disrupts physical barriers and releases nutrients, creating permissive and heterogeneous microenvironments that facilitate opportunistic colonization and variable microbial succession and therefore satisfies Anna Karenina principle (Arnault et al., 2023). However, it is also possible that the enriched bacterial and fungal taxa, once established, may further accelerate rot progression through enzymatic degradation and metabolic interactions. Therefore, the increasing heterogeneity of microbial communities with disease severity reflects a feedback loop in which host tissue breakdown and microbial proliferation reinforce each other, rather than a single deterministic pathogenic pathway. Importantly, the concurrent shifts in bacterial and fungal communities also indicate the possibility of cross-kingdom (bacteria-fungi) cooperation in onion tissue degradation. Fungi are well recognized for initiating cell-wall breakdown through secretion of cellulolytic and pectinolytic enzymes, thereby releasing soluble substrates that can be exploited by bacteria (Lattanzio et al., 2006; Deveau et al., 2018). Such fungal–bacterial interactions have been shown to enhance disease progression in plant systems by accelerating nutrient cycling and modifying local microenvironments. The coordinated succession patterns observed in this onion rot studysupport a model in which host tissue decay, microbial dysbiosis, and inter-microbial cooperation operate as an interconnected feedback loop rather than as isolated processes.

This study also examined the composition and succession of fungal communities associated with healthy and rotten onion bulbs. A clear restructuring of the onion bulb mycobiome was observed with differential rot severity, highlighting strong tissue specificity and stage-dependent fungal succession. Overall, 53 fungal OTUs were detected across the nine onion bulb samples, with very low fungal colonization observed in healthy bulbs, and this level of diversity is comparable to the 40 fungal OTUs reported by Yurgel et al. (2018) in their onion microbiome study. Overall, healthy bulbs harbored a very limited fungal community, particularly in the central and outer scale tissues, indicating that intact bulb tissues are largely resistant to fungal colonization. The comparatively higher fungal presence in the neck tissue of healthy bulbs (Shannon index = 0.53), dominated by *Aspergillus*, contrasted with the markedly lower fungal sequences observed in the outer scales (Shannon index = 0.10) and central tissues (Shannon index = 0.04). The transition from healthy to mildly rotten bulbs was marked by the emergence of several fungal genera that were completely absent in healthy tissues, particularly enrichment was more apparent in the neck region. The appearance and dominance of *Meyerozyma*, *Blastobotrys*, and *Penicillium* in mildly rotten neck tissue suggests that early tissue degradation creates favorable niches for opportunistic yeasts and filamentous fungi. Although *Penicillium* is widely reported as a pathogen of storage onion bulbs (Çakır and Maden, 2015), the yeast like ascomycetes fungi *Meyerozyma* and *Blastobotrys* are not reported as onion bulb pathogens, however, *Meyerozyma* enrichment in rotten bulbs has also been observed in other amplicon-based studies of diseased onion tissues (Yurgel et al., 2018), which suggests a recurrent association of this fungal genus with bulb decay rather than a sporadic occurrence in this study. The neck tissue remained the primary site of fungal colonization in mildly rotten bulbs, exhibiting a higher Shannon diversity index (MRB-NT = 1.74) than the outer scales (MRB-OS = 0.54) and central tissue (MRB-CT = 0.52). Since maximum fungal activity occurs through the neck tissue, the importance of proper curing and complete neck closure is essential to ensure better storability, as incomplete neck closure resulting from improper curing has been reported to predispose bulbs to bacterial and fungal ingression, resulting in faster bulb spoilage (Schroeder and Du Toit, 2010; Vahling-Armstrong et al., 2016). As rot progressed to the severe stage, a pronounced dominance of fermentative yeasts, particularly *Pichia* and *Kazachstania*, was observed across all bulb tissues, including the central tissue. This marked shift toward yeast-dominated communities indicates a major ecological transition within the bulb microenvironment, favoring fermentative processes. The predominance of *Kazachstania* has been shown in the fermentative onion foods along with many other yeast genera like *Candida,* and *Saccharomyces* (Cheng et al., 2014). Since the bacterial community analysis also revealed a concurrent increase in fermentative and oxidative bacterial genera in severely rotten bulb tissues, such as *Lactobacillus* (33.2%) and *Acetobacter* (16.1%), together with fermentative fungal genera, these patterns indicate that onion bulb rotting progresses predominantly through fermentation-driven processes. Severe tissue maceration, oxygen limitation, and accumulation of fermentable substrates likely favor yeast proliferation over filamentous fungi at this stage. The overwhelming abundance of *Pichia* in severely rotten tissues suggests its strong competitive advantage under advanced decay conditions and points to its potential role in driving secondary fermentation processes that accelerate bulb softening and tissue collapse. Previous studies on spoiled vegetable microbiota have shown that the production of extracellular hydrolases like cellulases, xylanases, and pectinases (pectin lyase and polygalacturonase), protease, amylase, and lipases correlates with the severity of rot, with pectinases often playing a dominant role in cell wall breakdown (Lee et al., 2013). Several bacterial taxa enriched in decayed onion tissues (e.g., *Acinetobacter*) have been reported to produce pectinase activity on plant substrates, supporting the possibility that similar hydrolytic mechanisms contribute to tissue degradation in onion bulb rot (Bhardwaj, 2023). Similarly, many species of fungal genera *Aspergillus* enriched in rotten tissue are also the well-documented producer of hydrolases like cellulase, pectinase, *β*-glucosidases, and xylanases, which can break down structural polysaccharides in plant tissues (Rehman et al., 2014; Matsuzawa, 2024). Other fungal genera enriched in rotten bulb tissues included *Penicillium*, several species of which are known to produce extracellular plant cell wall–degrading enzymes such as pectinases and cellulases. Enrichment of hydrolase-producing microorganisms in rotten tissues suggests their potential involvement in onion bulb tissue degradation through cell wall polysaccharide weakening and subsequent tissue maceration. The increased abundance of bacterial genera such as *Lactobacillus* and *Gluconobacter*, and fungal genera such as *Pichia* and *Kazachstania*, in advanced rot stages (SRB) suggests that their role in bulb decay may be mediated through microenvironmental changes, such as acidification, rather than direct hydrolase action, as these genera are primarily associated with fermentative metabolism rather than the production of cellulases or pectinases. This study underscores the need to reconsider onion bulb rotting as a dynamic process of microbial succession rather than a disease driven by a single pathogen. Our findings reveal that several bacterial and fungal genera which are classically not recognized as major postharvest pathogens of fruits and vegetables becomes abundant during bulb rot progression, thereby highlighting their potential role in bulb decay. The present study adopts a tissue- and stage-resolved sampling strategy to capture microbiome restructuring associated with progressive onion bulb rot. By focusing on representative bulbs across defined health states and dissecting distinct tissues, the analysis emphasizes qualitative shifts in dominant taxa, community composition, and ecological transitions along the bulb rot stages. Such an approach is particularly suited to generating information of niche-specific microbial colonization, potential pathogen reservoirs, and tissue-dependent host–microbe interactions, while increased biological replication would further strengthen quantitative inference, the patterns observed here provide a first framework for understanding tissue-specific microbial assembly during bulb deterioration and serve as a basis for future studies incorporating larger sample sizes for predictive modeling. Elucidating the ecological functions, growth requirements (including pH and temperature optima), and interaction dynamics of these dominant microbial taxa will be critical for developing targeted and stage-specific strategies to slow or prevent bulb rotting during storage and postharvest handling.

## 5 Conclusion

This study characterized the bacterial and fungal communities associated with healthy and rotten onion bulbs across distinct bulb tissues. High-throughput amplicon sequencing revealed a structured succession of both bacterial and fungal communities characterized by an initial expansion of taxonomic diversity during early decay, followed by a pronounced dominance of a few specialized taxa at severe rot stages. The increased abundance of bacterial genera such as *Lactobacillus*, *Acetobacter*, *Gluconobacter*, and *Carnimonas*, together with yeasts including *Pichia* and *Kazachstania*, underscore their potential roles in driving bulb tissue degradation although many of these genera are not classically described as common pathogens of onion bulb. Further functional and culture-based studies will be required to clarify the specific roles of these taxa in rot development and to translate these insights into effective postharvest management strategies. Tissue-specific analyses further revealed the bulb neck tissue as the primary fungal succession zone, likely facilitating initial colonization and subsequent inward spread during progression of rotting. Overall, these findings highlight onion bulb rotting as a microbiome-driven, successional process shaped by bulb tissue niche and rot severity. By elucidating the rot-stage and tissue-specific changes in bacterial and fungal community compositions, this study advances our understanding of microbial processes associated with postharvest bulb decay.

## Data Availability

The datasets presented in this study can be found in online repositories. The names of the repository/repositories and accession number(s) can be found in the article/Supplementary material.
