# Supplementary material for: Microbial succession and tissue-specific restructuring of bacterial and fungal communities during post-harvest onion bulb rotting
Source: Front Microbiol. 2026 Feb 20;17:1776996. doi: 10.3389/fmicb.2026.1776996 (PMC12963344; doi:10.3389/fmicb.2026.1776996)
Supplement: Supplementary file 1 [file Data_Sheet_1.zip › Datasheet 1.docx]

**Supplementary Table: S1** Month-wise Average Temperature (Max & Min), Relative Humidity (8.45 AM and 2.45 PM, Indian Standard Time) and Rainfall at onion bulb storage site, Rajgurunagar, Pune, India.

| Month | T max (°C) | T min (°C) | RH (%)  8:45 AM | RH (%) 2:45 PM | Total rainfall (mm) |
| --- | --- | --- | --- | --- | --- |
| April-2024 | 39.25±0.7 | 20.73± 3 | 65.32± 6.7 | 42.11±6.7 | 0 |
| May-2024 | 38.09±2 | 22.91±2.1 | 71.21± 7.6 | 49.6± 7.2 | 0 |
| June-2024 | 32.96+2.1 | 23.06±0.9 | 83.56±5.7 | 66.86±14.6 | 105 |
| July-2024 | 28.15+1.8 | 22.78+0.8 | 90.45±5 | 81.4±8.3 | 237 |
| August-2014 | 28.75+2.3 | 22.04+0.7 | 90.13±8.5 | 79.31±8.5 | 231 |
| September-2024 | 29.61+1.7 | 21.56+1.5 | 86.51±3.9 | 73.46±9.5 | 67 |
| October-2024 | 32.36+1.4 | 21.45+1.5 | 86.06±5.8 | 65.45±9.7 | 77 |

**Supplementary Table S2:** Visual and sensory characteristics used to categorize onion bulbs into healthy (HB), mildly rotten (MRB), and severely rotten (SRB) stages

| Visual/sensory Characters | Healthy bulb (HB) | Mildly rotten bulb  (MRB) | Severely rotten bulb  (SRB) |
| --- | --- | --- | --- |
| Lesions/tissue damage | Completely absent | Present mainly confined to upper scales, not reaching to central scales | Advanced, widespread decay; extensive involvement of internal tissues including central scales |
| Tissue firmness/Consistency | Complete bulb tissue intact and firm | Partial softening of affected only the affected area whereas the unaffected tissue remained firm and structurally intact. | Extensive softening with tissue maceration or liquefaction; loss of normal tissue structure. |
| Color | Uniform color, no intense discoloration of bulb | Slight discoloration (light brown to yellow color confined to effected area) | Pronounced discoloration (dark brown/black) affecting large portions of the bulb. |
| Odor | No unusual order | Mild or early-stage foul or fermentative odor. | Strong, offensive putrid odor |
| Neck region | Completely intact | Slightly softening of neck region. | Neck region collapsed, wet, or extensively degraded. |
| Tissue exudation | Complete absent | No tissue exudation, only slight slippery outer scales | Excessive moisture, ooze, or slimy exudate from bulb with visible tissue liquefaction |

**Supplementary Table S3:** Sample-wise summary of raw read (16S rRNA V3–V4) paired end reads with SRA Accessions and ASV Counts

**Supplementary Table S4:** Sample-wise summary of raw ITS paired end reads with SRA Accessions and ASV Counts

| Sample name | Raw 16S reads | | NCBI-SRA accession | Number of ASV’s | Frequency of ASVs  detected in each sample |
| --- | --- | --- | --- | --- | --- |
|  | Forwards Reads (R1) | Reverse Reads (R2) |  |  |  |
| HB-NT | 106558 | 106558 | SRR35529117 | 172 | 63657 |
| HB-CT | 103026 | 103026 | SRR35529116 | 183 | 59365 |
| HB-OS | 92498 | 92498 | SRR35529115 | 161 | 55,100 |
| MRB-NT | 97378 | 97378 | SRR35529114 | 278 | 47,719 |
| MRB-CT | 97256 | 97256 | SRR35529113 | 255 | 53,081 |
| MRB-OS | 85283 | 85283 | SRR35529112 | 224 | 45,679 |
| SRB-NT | 92507 | 92507 | SRR35529111 | 144 | 33831 |
| SRB-CT | 86374 | 86374 | SRR35529110 | 238 | 52,494 |
| SRB-OS | 93840 | 93840 | SRR35529109 | 210 | 55,132 |
| Total reads | 854720 | 854720 |  |  | 466,058 |

| Sample name | **Raw ITS reads** | | NCBI-SRA accession | Number of ASV’s | Frequency of ASVs  detected in each sample |
| --- | --- | --- | --- | --- | --- |
|  | Forwards Reads (R1) | Reverse Reads (R2) |  |  |  |
| HB-NT | 130671 | 130671 | SRR35529485 | 53 | 99070 |
| HB-CT | 136847 | 136847 | SRR35529483 | 47 | 96979 |
| HB-OS | 168518 | 168518 | SRR35529484 | 52 | 124718 |
| MRB-NT | 123193 | 123193 | SRR35529482 | 91 | 113394 |
| MRB-CT | 88278 | 88278 | SRR35529480 | 54 | 64690 |
| MRB-OS | 126673 | 126673 | SRR35529481 | 44 | 94269 |
| SRB-NT | 152089 | 152089 | SRR35529479 | 54 | 140904 |
| SRB-CT | 91482 | 91482 | SRR35529477 | 40 | 81795 |
| SRB-OS | 90146 | 90146 | SRR35529478 | 58 | 72374 |
| Total | 1107897 | 1107897 |  | 198 | 888193 |


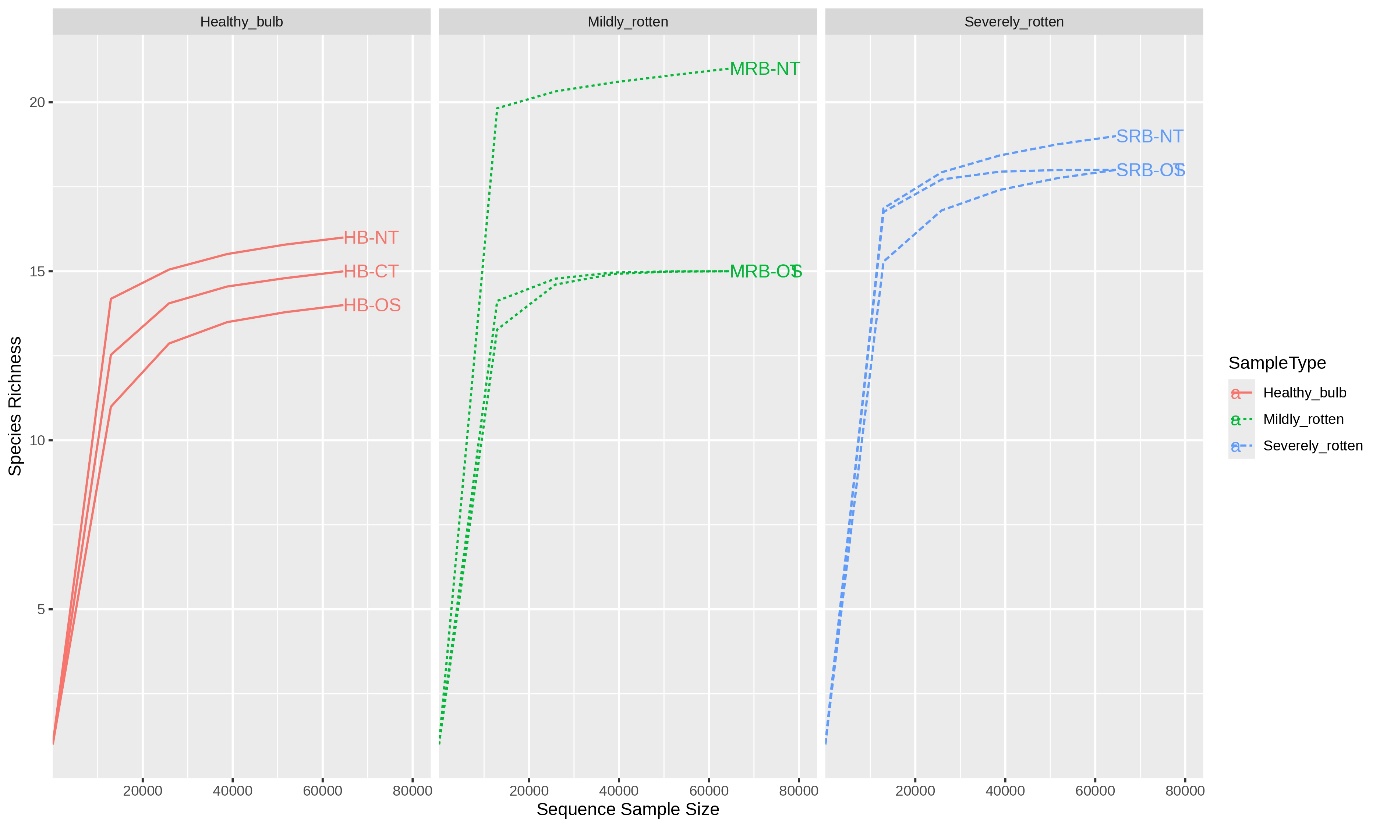

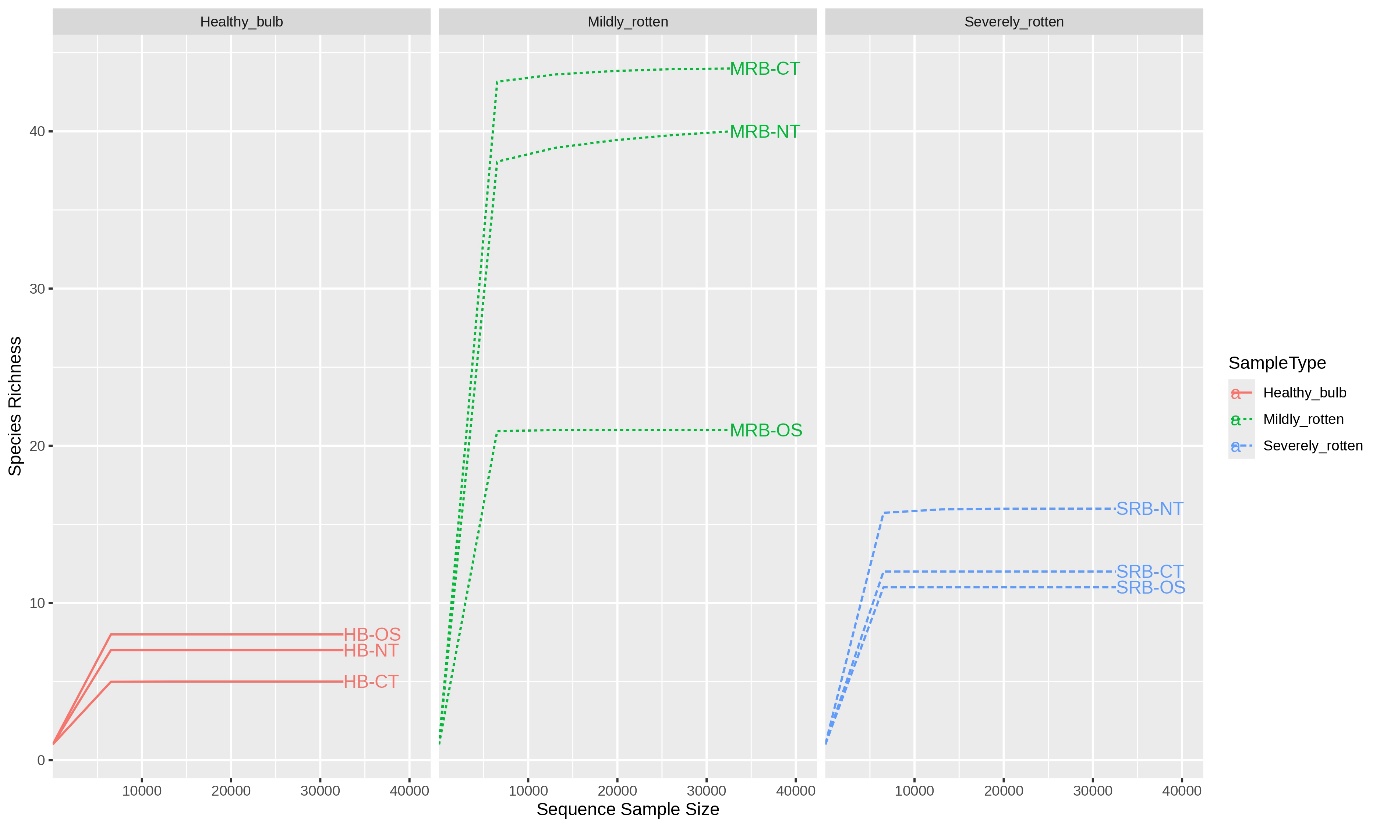


Supplementary Figure: S1 Rarefaction curves illustrating sequencing depth and species richness for (A) bacterial and (B) fungal microbiome communities across samples. Curves represent the number of observed taxa as a function of sequencing reads, demonstrating adequate sampling depth and allowing comparison of bacterial and fungal diversity after rarefaction to the minimum sequencing depth.

B

A
